# Impact of prehospital medical evacuation (MEDEVAC) transport time on combat mortality in patients with non-compressible torso injury and traumatic amputations: a retrospective study

**DOI:** 10.1186/s40779-018-0169-2

**Published:** 2018-06-30

**Authors:** Joseph K. Maddry, Crystal A. Perez, Alejandra G. Mora, Jill D. Lear, Shelia C. Savell, Vikhyat S. Bebarta

**Affiliations:** 1 US Air Force En route Care Research Center 59th MDW/ST, Chief Scientist’s Office –US Army Institute of Surgical research, JBSA Ft. Sam Houston, San Antonio, TX USA; 2 Department of Emergency Medicine, San Antonio Military Medical Center, JBSA Ft. Sam Houston, San Antonio, TX USA; 3 Department of Emergency Medicine, University of Colorado School of Medicine, Aurora, CO USA

**Keywords:** Transport time, Non-compressible torso injury, Traumatic amputation, Combat

## Abstract

**Background:**

In combat operations, patients with traumatic injuries require expeditious evacuation to improve survival. Studies have shown that long transport times are associated with increased morbidity and mortality. Limited data exist on the influence of transport time on patient outcomes with specific injury types. The objective of this study was to determine the impact of the duration of time from the initial request for medical evacuation to arrival at a medical treatment facility on morbidity and mortality in casualties with traumatic extremity amputation and non-compressible torso injury (NCTI).

**Methods:**

We completed a retrospective review of MEDEVAC patient care records for United States military personnel who sustained traumatic amputations and NCTI during Operation Enduring Freedom between January 2011 and March 2014. We grouped patients as traumatic amputation and NCTI (AMP+NCTI), traumatic amputation only (AMP), and neither AMP nor NCTI (Non-AMP/NCTI). Analysis was performed using chi-squared tests, Fisher’s exact tests, Cochran-Armitage Trend tests, Shapiro-Wilks tests, Wilcoxon and Kruskal-Wallis techniques and Cox proportional hazards regression modeling.

**Results:**

We reviewed 1267 records, of which 669 had an injury severity score (ISS) of 10 or greater and were included in the analysis. In the study population, 15.5% sustained only amputation injuries (n=104, AMP only), 10.8% sustained amputation and NCTI (n=72, AMP+NCTI), and 73.7% did not sustain either an amputation or an NCTI (n=493, Non-AMP/NCTI). AMP+NCTI had the highest mortality (16.7%) with transport time greater than 60 min. While the AMP+NCTI group had decreasing survival with longer transport times, AMP and Non-AMP/NCTI did not exhibit the same trend.

**Conclusions:**

A decreased transport time from the point of injury to a medical treatment facility was associated with decreased mortality in patients who suffered a combination of amputation injury and NCTI. No significant association between transport time and outcomes was found in patients who did not sustain NCTI. Priority for rapid evacuation of combat casualties should be given to those with NCTI.

**Electronic supplementary material:**

The online version of this article (10.1186/s40779-018-0169-2) contains supplementary material, which is available to authorized users.

## Background

In combat operations, patients with traumatic injuries require urgent clinical care and expeditious evacuation to improve survival [1]. In recent wars in Iraq and Afghanistan, prehospital providers such as ground medics and aeromedical evacuation (AE) teams were often the first responders. Aeromedical evacuation platforms such as MEDEVAC allow for urgent evacuation to medical treatment facilities (MTF) that can provide the more complex, necessary lifesaving interventions that are not otherwise possible at point of injury (POI) or en route. Transport times may vary depending on environmental factors and the ability to land in combatant locations [2]. Urgent response and transport can be delayed due to tactical issues, which may interfere with timely life-saving care. Previous studies have shown that long transport times are associated with increased morbidity and mortality [3]. There are limited data on the influence of transport time on patient outcomes with specific types of injury.

Compared to civilian trauma, combat-related injuries are unique due to the explosive weapons and high velocity projectiles used in war. Blast related injuries were the leading mechanism of injury sustained during recent military conflicts [4–6]. As a result, traumatic extremity amputation is common among the combat injured. Between 2000 and 2011, over 1600 cases of military traumatic amputation were reported [7]. At the POI, ground medics or fellow combatants applied tourniquets to prevent hemorrhage; however, swift evacuation for surgical hemorrhage control may influence a patient’s long-term outcome.

Non-compressible torso hemorrhage (NCTH) consists of those injuries resulting in intrathoracic or intra-abdominal hemorrhage that cannot be controlled with manual pressure. NCTH has been defined as vascular disruption from 1 or more of the following anatomic categories: the thoracic cavity, solid organ injury > grade 4 (liver, kidney, spleen), named axial torso vessel, and pelvic fracture with ring disruption. [8]. NCTH is the most common cause of potentially survivable death in both military and civilian trauma [8–13]. The mortality rate for combat NCTH is over 85%, and almost 90% of deaths occur before arrival to an MTF [12]. Prehospital management of non-compressible torso injury (NCTI) presents the greatest opportunity to improve survival from combat trauma. Understanding the effect of prehospital transport time may assist in improving the management of this highly lethal injury pattern.

While previous research has demonstrated a direct relationship between transport time and combat mortality, whether or not decreased evacuation time for specific wartime injuries confers a benefit is not yet known [14]. The primary objective of this study was to determine the impact of the duration of time from the initial request for medical evacuation to arrival at an MTF on morbidity and mortality through thirty days after injury, in those casualties with traumatic extremity amputation and non-compressible torso injury.

## Methods

We obtained approval from the Wilford Hall Ambulatory Surgical Center Institutional Review Board (IRB) and conducted this study under the approved protocol. We completed a retrospective review of MEDEVAC patient care records (PCRs) for United States (US) military personnel who sustained traumatic amputations and NCTI in the Operation Enduring Freedom (OEF) Theater of Operations between January 2011 and March 2014. This study was an additional (or extension) analysis performed on a dataset (convenience sample of consecutive records) previously compiled [15]. In the previous study, we excluded PCRs of casualties who were documented to be non-survivors at the POI or were transported to an MTF solely to be pronounced dead. This was to exclude the casualties who did not receive any interventions and for whom transport time would not have made a difference in outcome.

To identify the patient population of interest, we queried the Department of Defense Trauma Registry (DoDTR) with specified International Classification of Diseases (ICD)-9/10 (ICD-9/10) codes and Abbreviated Injury Score (AIS) codes (Additional file 1) [12]. The retrieved list of patients was matched with our study electronic database containing data from abstracted PCRs. Patient data from the POI to the first MTF was abstracted from PCRs by trained research team members and entered into an electronic database (Microsoft Excel 2010, Redmond, WA). Data points included demographics, injury description, provider type, procedures, medications administered, clinical events, analgesics administered, and in-theater survival. Transport time was estimated by using the time stamp of the 9-Line call (request for medical evacuation) to time of arrival at the first MTF. The 9-Line call time was the most consistently available (98%) and highly correlated (R-value: 0.9757; 95% CI: 0.9729-0.9782) with injury time. Clinical events were identified from provider narrative and descriptions of events documented in the PCR. Missing or unavailable data were reconciled using the Theater Medical Data System (TMDS). We implemented a quality assurance (QA) process to ensure consistency among abstractors, and to include secondary abstractor review and reconciliation of 100% of records [15].

In our study database, we included an injury severity score (ISS) and maximum AIS for each of the six body regions provided by DoDTR. For this study, we excluded casualties with an ISS less than 10 to focus on comparable study groups and concentrate on severely injured casualties who would have benefited from shorter transport. The dataset also included supplemental outcome data such as vital signs, complications, ventilator days, intensive care unit (ICU) days, hospital days, mortality, and disposition at discharge from each MTF and up to 30 days. For statistical analysis, we grouped patients as traumatic amputation and NCTI (AMP+NCTI), traumatic amputation only (AMP), and neither AMP nor NCTI (Non-AMP/NCTI). No patients in our study had NCTI without AMP. We also binned patients using transport time intervals: <30 min, 30-60 min, and >60 min. We evaluated categorical data using chi-squared and, as appropriate, Fisher’s exact tests. The Cochran-Armitage Trend test was applied to evaluate the association between survival rates and transport-time intervals. Proportions were reported as percentages along with 95% confidence interval. Following the Shapiro-Wilks test and normality plot assessments, we compared continuous variables using Wilcoxon and Kruskal-Wallis techniques. Regression analyses were limited due to low mortality; thus, we performed Cox proportional hazards regression modeling for time to discharge from ICU and hospital days. Analyses were conducted using JMP version 13 (SAS Institute Inc., Cary, NC).

## Results

We reviewed 1267 PCRs, of which 669 had an ISS of 10 or greater and were included in the analysis. In this study, 15.5% sustained only amputation injuries (n=104, AMP only), 10.8% amputation and non-compressible torso injuries (n=72, AMP+NCTI), and 73.7% did not sustain either an amputation or a non-compressible torso injury (n=493, Non-AMP/NCTI). Of the 176 patients with AMP, 40.9% (n=72) also had NCTI. Most injured patients were male (98.8%) with a median age of 24 years old, and these proportions were not different among the groups (Table 1). With a median transport time of 36 min, there was no significant difference in elapsed time from POI to MTF among the three groups (p=0.7793). Casualties were transported to a Role 2 (52.0%) or Role 3 (48.0%) facility. Medical capabilities increase with the higher Role designation. Role 2 MTFs have ability to perform damage control surgery and advanced resuscitation, but have limited holding ability. The Role 3 MTF is a field hospital with expanded surgical and imaging capabilities as well as capacity to hold patients. The predominant mechanism of injury was explosion (72.3%) followed by penetrating injuries (26.5%). AMP+NCTI patients were more severely injured (median ISS of 33), followed by AMP and Non-AMP/NCTI.

**Table 1:** Descriptive summary of study population: US casualties transported from point-of-injury to MTF via MEDEVAC (%, 95 CI (count) or median [IQR])

Item	All (*n =* 669)	AMP + NCTI (*n =* 72)	AMP (*n =* 104)	Non-AMP/NCTI (*n =* 493)	*P* value
Male	99, 98–100 (661/665)	100, 95–100 (72/72)	100, 96–100 (103/103)	99, 98–100 (486/490)	0.2935
Age (year)	24 [22–28]	23 [21–27]	24 [21–27]	24 [22–28]	0.1128
Injury to MTF (min)	41 [31–56]	34 [28–45]	34 [27–46]	44 [33–59]	< 0.0001
9-Line to MTF (min)	36 [29–47]	32 [25–40]	32 [27–44]	38 [30–51]	< 0.0001
Injury description*					
Blast	72, 69–76 (484/669)	100, 95–100 (72/72)	95, 89–98 (99/104)	63, 59–68 (313/493)	< 0.0001
Penetrating	26, 23–30 (177/669)	0, 0–5 (0/72)	4, 2–9 (4/104)	35, 31–39 (173/493)	< 0.0001
Blunt	1, 0.6–2 (8/669)	0, 0–5 (0/72)	1, 0.2–5 (1/103)	1, 0.6–3 (7/493)	0.7749
ISS (score)	17 [12–27]	33 [25–40]	18 [14–27]	17 [12–24]	< 0.0001
GCS of 3	3, 2–5 (17/552)	2, 0.2–8 (1/65)	3, 1–9 (3/89)	3, 1–6 (13/398)	0.7042
Head injury (AIS of head ≥2)	65, 56–74 (240/369)	74, 60–85 (32/43)	62, 50–73 (38/61)	64, 52–75 (170/265)	0.1169
Prehospital hypotension (SBP < 90 mmHg)	25, 21–29 (117/472)	47, 35–60 (26/55)	27, 18–37 (22/82)	21, 17–25 (69/335)	0.0015
30-day mortality	5, 3–7 (31/662)	8, 4–17 (6/72)	4, 2–10 (4/103)	4, 3–7 (21/487)	0.3045

*AMP+NCTI* Traumatic amputation and non-compressible torso injury, *AMP* Traumatic amputation only, *Non-AMP/NCTI* Neither traumatic amputation nor non-compressible torso injury, *CI* Confidence interval, *IQR* Interquartile range, *MTF* Medical Treatment Facility, *ISS* Injury Severity Score, *GCS* Glasgow Coma Scale, *AIS* Abbreviated injury scale, *SBP* Systolic blood pressure; *Blast, penetrating, and blunt are mutually exclusive

**Fig. 1:**
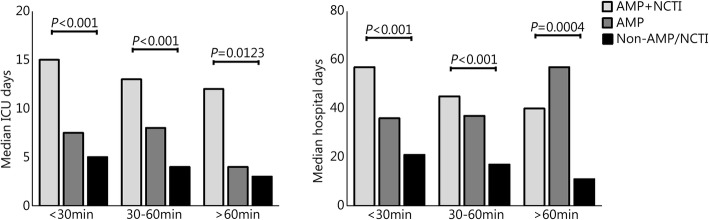
ICU stay and hospital stay outcomes following prehospital transport of study groups. <30 min vs 30-60 min vs >60 min, Median ICU days: *P*=0.1884 in AMP+NCTI; *P*=0.3479 in AMP; *P*=0.0667 in Non-AMP/NCTI Median hospital days: *P*=0.2412 in AMP+NCTI; *P*=0.3704 in AMP; *P*=0.0036 in Non-AMP/NCTI. AMP+NCTI. Traumatic amputation and non-compressible torso injury; AMP. Traumatic amputation only; Non-AMP/NCTI. Neither traumatic amputation nor non-compressible torso injury

**Fig. 2:**
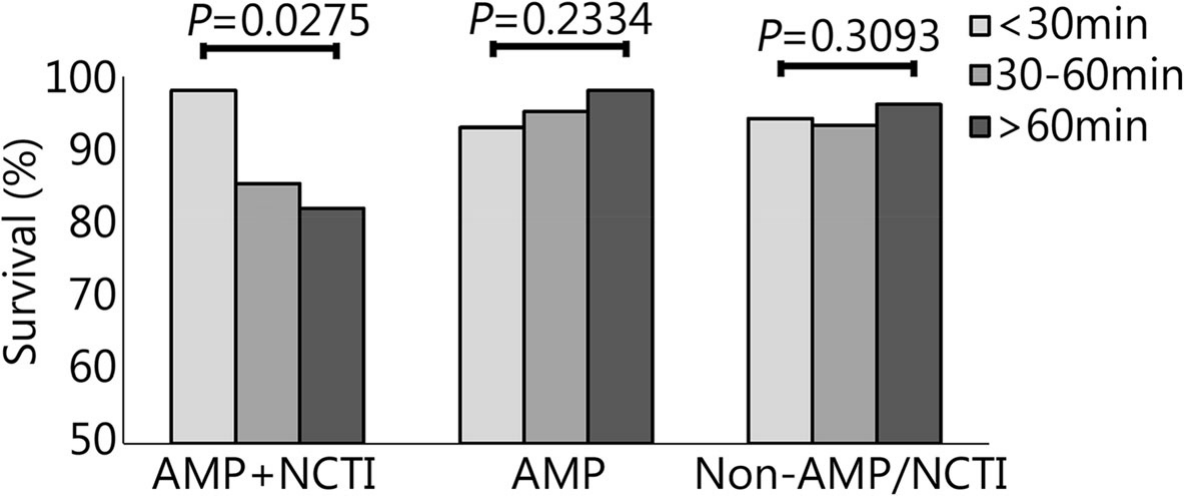
Study group percent survival by transport time. AMP+NCTI. Amputation and non-compressible torso injury; AMP. Traumatic amputation only; Non-AMP/NCTI. Neither traumatic amputation nor non-compressible torso injury; min. Minutes

Evaluating study injury groups by transport time, the median ISS was higher in AMP+NCTI at each time interval (Table 2). AMP and AMP+NCTI were more likely to be transported to a Role 3 (74.5% and 66.2%, respectively) compared to Non-AMP/NCTI (39.7%, P<0.0001). The Non-AMP/NCTI group was least likely to have received tourniquets, blood products, intravenous (IV) fluids, or an airway procedure during prehospital transport. Likewise, the Non-AMP/NCTI group had the least number of prehospital procedures performed (Table 3). When comparing by transport time groups, casualties were transported to a Role 2 (50.6%, <30 min; 51.9%, 30-60 min; 56.3%, >60 min) or Role 3 facility (49.4%, <30 min; 48.1%, 30-60 min; 43.8%, >60 min) in equal proportions (*P*=0.7411). Blood product administration was more likely in the 30-60 min (10.0%) group compared to <30 min (4.8%) or >60 min (4.2%, *P*=0.0339) groups. We did not note any other incidence rate differences in prehospital procedures performed between the study transport-time groups. AMP+NCTI had more days spent in the ICU and in the hospital (Fig. 1). AMP+NCTI had the highest mortality (16.7%) with transport time greater than 60 min. While the AMP+NCTI group had decreasing survival with longer transport times, AMP and Non-AMP/NCTI did not exhibit the same trend (Fig. 2).

**Table 2:** ISS by injury type and transport time groups (n, median (IQR))

Duration	AMP+NCTI (n=72)	AMP (n=104)	Non-AMP/NCTI (n=493)	*p* value
<30 min	33 (24-41) (27)	18 (14-26) (38)	17 (12-26) (122)	<0.0001
30-60 min	33 (24-43) (39)	19 (14-27) (59)	17 (11-22) (312)	<0.0001
>60 min	29 (28-38) (6)	21 (17-27) (7)	14 (11-22) (59)	0.0003

*ISS* Injury severity score, *AMP+NCTI* Traumatic amputation and non-compressible torso injury, *AMP* Traumatic amputation only, *Non-AMP/NCTI* Neither traumatic amputation nor non-compressible torso injury, *IQR* Interquartile range

**Table 3:** Prehospital interventions performed (%, 95% CI (count) or median [IQR])

Item	All *n*=669	AMP+NCTI *n*=72	AMP *n*=104	Non-AMP/NCTI *n*=493	*p*-value
Tourniquets	51, 47-55 (342/669)	100, 95-100 (72/72)	91, 84-95 (95/104)	35, 31-40 (175/493)	<0.0001
IV fluids	54, 50-58 (360/669)	62, 54-75 (47/72)	72, 63-80 (75/104)	48, 44-53 (238/493)	<0.0001
Blood	8, 6-10 (53/669)	17, 10-27 (12/72)	27, 19-36 (28/104)	3, 2-4 (13/493)	<0.0001
Chest needle	4, 3-6 (27/669)	3, 1-10 (2/72)	1, 0.2-5 (1/104)	5, 3-7 (24/493)	0.0914
Any airway	58, 55-62 (390/669)	78, 67-86 (56/72)	72, 63-80 (75/104)	53, 48-57 (259/493)	<0.0001
Chest seal	5, 4-7 (35/669)	100, 95-100 (72/72)	2, 1-7 (2/104)	7, 5-9 (33/493)	0.0018
Number of prehospital interventions	2 [1-4]	4 [2-4]	4 [2-4]	2 [1-3]	<0.0001

*AMP+NCTI* Traumatic amputation and non-compressible torso injury, *AMP* Traumatic amputation only, *Non-AMP/NCTI* Neither traumatic amputation nor non-compressible torso injury, *CI* Confidence interval, *IQR* Interquartile range, *IV fluids* Intravenous fluids

In proportional hazard models, the AMP and Non-AMP/NCTI groups combined were more likely to discharge from the ICU more quickly (risk ratio 2.29; 95CI: 1.79-2.97) compared to the AMP+NCTI group (*P*<0.0001). We had similar findings in models of time to hospital discharge. AMP and Non-AMP/NCTI combined were more likely to discharge from the hospital more quickly (risk ratio 2.5; 95CI: 1.94-3.26) than the AMP+NCTI group (*P*<0.0001).

Adjusting for injury group and ISS, patients with a transport time interval >60 min were likely to discharge from the ICU more quickly (risk ratio 1.43; 95CI: 1.03-1.99) compared to <30 min transports (*P*=0.0329). Additionally, while neither tourniquet alone nor blood alone decreased risks, patients who had any combination of tourniquet and blood product administration prehospital were likely to discharge from the ICU more quickly (risk ratio 2.71; 95CI: 1.03-7.1; *P*=0.0425).

In time-to-hospital-discharge models, after adjusting for injury group and ISS, patients with transport time interval 30-60 and >60 min were likely to discharge from the hospital more quickly (risk ratio 1.34; 95CI: 1.12-1.61; *P*=0.0016 and 1.62; 95CI: 1.16-2.23; *P*=0.0053; respectively) compared to <30 min transports. Prehospital procedures did not reduce the risk of a longer hospital stay.

### Additional sub analysis

Evaluating moderate-to-severe head injury with concomitant amputation(s), there was no significant in-theater mortality difference (2.9% versus 0.0%; *P*=1.000). A larger sample size with a greater mortality rate may yield different results. However, decreased level of consciousness (GCS of 3) combined with hypotension (SBP<90) was associated with increased odds of mortality (4.17 ;95CI :1.84-9.45; *P*=0.0006). Hypotension alone did not increase odds of mortality.

Subsequently, we assessed the influence of concomitant upper and lower limb amputations. Sustaining both upper and lower limb amputations presented with the highest in-theater mortality (combined, 11.4%; lower amputation, 2.9%; upper amputation, 0.0%; *P*=0.0288). Faster evacuation times did not confer a survival benefit to patients who sustained combined upper and lower limb amputations (*P*=0.7541).

## Discussion

Our results demonstrate a statistical association between shorter transport times and AMP+NCTI survival; however, transport time was not associated with outcomes in those patients with isolated extremity amputations. Our results may guide future evacuation prioritization based on those who stand to gain the greatest benefit from expeditious evacuation or from far forward prehospital interventions when rapid evacuation is not feasible.

While previous civilian and military studies have evaluated the impact of the “golden hour,” our study evaluated the impact of transport time during a unique time in military medical history. On June 15, 2009, Secretary of the U.S. Department of Defense, Robert M. Gates, established a policy that the time from medical evacuation request until the injured patient arrived at a treatment facility should be less than 60 min [16]. Our study evaluates the impact of medical transport times over a narrow timeframe of less than 90 min. Our data are also unique given that our patient sample occurred after the widespread use of tourniquets, the forward deployment of blood product administration, the increased utilization of paramedics and nurses for medical evacuation, and the increased transport of patients directly to a combat support hospital instead of to a forward surgical team [14]. These interventions, aimed at decreasing preventable combat mortality, likely altered the impact of medical evacuation times on patient outcomes.

Despite recent advancement in prehospital combat casualty care, our study reflects the continued importance of minimizing prehospital evacuation times in patients with NCTI. Previous autopsy-based studies have determined that most potentially preventable combat deaths occurred due to exsanguination from the torso [17, 18]. Our finding of a direct association between transport time and mortality in NCTI supports these results. However, civilian literature has demonstrated mixed results, with most studies finding no significant association between transport time and mortality in patients with thoracoabdominal injury [19]. The generalization of these findings to the civilian population is questionable, as injuries from explosive devices or high velocity rifles are uncommon in the civilian environment but account for most injuries in our dataset.

Our study found no association between transport time and mortality in patients who suffered AMP without NCTI. This may be because our patient sample was taken after the widespread implementation of rapid tourniquet use. Rapid control of hemorrhage with tourniquet application likely allows for survival during extended evacuation. While previous studies have found that a considerable number of combatants died from extremity exsanguination, this was prior to the widespread adoption of easily and rapidly applied tourniquets [17, 18]. Studies conducted later during the conflict in Afghanistan found high rates of tourniquet use, which likely accounts for our findings [20].

Previous studies evaluated the impact of medical evacuation capabilities on mortality in relation to AIS and ISS. However, AIS and ISS are not tools available to combat medics at the time of injury. Medics are trained to routinely assess for NCTI, AMP, and other injuries. The results of our study can easily be disseminated to military medics and assist them in determining the appropriate level of triage and the urgency of rapid medical evacuation. NCTI may not have been identified or diagnosed by MEDEVAC providers; thus, expanded training to include use of ultrasound may be advisable for the continued optimization of care. Use of ultrasound has been fielded by medics and by other en route team members in the past. Several studies have supported the use of ultrasound by prehospital medics and non-clinical service members with minimal training [21–24]. In addition, ultrasound devices that are aided or have artificial intelligence such as the Butterfly [25] remove the learning curve for medics and provide results for clinical decision making. However, broad use and sustainment of skills is a challenge and an opportunity. Ultrasound is being used in military en route and austere settings, and newer off-the-shelf technology is making it easier for our medics. Furthermore, combining the findings of our study with previous research allows one to reasonably conclude that those patients with NCTI should receive the most advanced medical capabilities available.

Other studies have predominately focused on in-theater outcomes (approximately 24-72 hours after injury); conversely, our study evaluated the impact of transport time on 30-day outcomes. Shorter transport time could improve in-theater survival without impacting 30-day outcomes. We found an association between shorter transport times and 30-day mortality in patients with AMP + NCTI. Beyond mortality benefit, our study also found a direct relationship between transport time and duration of hospital and ICU stay in the AMP+NCTI group. Rapid transport of these patients has the potential to improve the patients’ quality of life and decrease utilization of medical resources. While increased equipment and personnel are necessary to decrease evacuation time, the cost may be offset by fewer hospital days and decreased utilization of inpatient medical resources .

Our study has several limitations. Most of our patients were evacuated within 1 hour. While a shorter evacuation time was not associated with decreased mortality in the AMP group, these results cannot be generalized to prolonged transport times (2+ hours). Lengthy transport times may still impact patients without NCTI in resource-limited areas of operation, such as the Pacific Ocean or Africa. Most of our patients suffered blast injuries, and our results may not be generalizable to those suffering from gun-shot wounds, aircraft crashes, and other forms of combat trauma. However, given the effectiveness and ease of the use of explosives, they are likely to remain a common source of combat casualties.

Furthermore, studies evaluating transport time are observational and not randomized; therefore, the potential for selection bias exists. Particularly, patients with more severe injury may be evacuated more rapidly as fellow combatants and medical personnel act with greater urgency in caring for this subgroup. Thus, those with the greatest injury and highest risk of death may be transported more quickly than those with less severe injuries (a basic premise of triage). In addition, shorter evacuation times along with the combat setting may have limited the opportunity for interventions such as blood product administration. However, there was no significant difference in ISS between the transport times, making this bias unlikely. Lastly, this study is reflective of combat-injured military members and may have limited generalizability in the civilian trauma populations.

Future research should evaluate the impact of rapid access to blood products, forward deployment of advanced medical providers and surgical capabilities, utilization of advanced en route care capabilities, and prehospital medical devices on the treatment of NCTI. The impact of transport time should also be evaluated in circumstances when these resources are available, as they may change the significance of evacuation time. As the military engages in operations resulting in significantly extended evacuation times of hours to days (i.e., Africa and the Pacific Ocean), military researchers and leaders will need to determine the effect of prolonged transport time on patient outcomes. Finally, studies evaluating the potential use of unmanned aerial vehicles or other tools to ensure rapid evacuation of combat casualties in resource-limited environments should be conducted.

Given that our study found that short evacuation times appear to confer the greatest benefit in those patients suffering from NCTI + AMP and other studies have found NCTI to be a leading cause of preventable combat mortality [7, 9], when feasible, evacuation times of patients with NCTI should remain under 30 min. In those circumstances where transport of NCTI patients from the POI to a Role 2/3 facility is not possible, rapid access to blood products, forward deployed advanced medical providers and advanced en route care capabilities, and/or resources for the control of NCTI may decrease mortality [1, 2, 13].

## Conclusion

A decreased transport time from the point of injury to the medical treatment facility was associated with decreased mortality in those patients who suffered a combination of an amputation injury and a non-compressible torso injury. No significant association between transport time and outcomes was found in patients who did not sustain a non-compressible torso injury. Priority for rapid evacuation of combat casualties should be given to those with non-compressible torso injury.

## Additional file

Additional file 1: List of ICD-9 and Abbreviated Injury Scale (AIS) codes used for query. (DOCX 20 kb)

## Abbreviation

AE: Aeromedical evacuation
AIS: Abbreviated injury score
AMP: Amputation
DoDTR: Department of Defense Trauma Registry
ICU: Intensive care unit
IRB: Institutional review board
ISS: Injury severity score
MTF: Medical treatment facility
NCTH: Non-compressible torso hemorrhage
NCTI: Non-compressible torso injury
OEF: Operation Enduring Freedom
PCR: Patient care record
POI: Point of injury
TMDS: Theater Medical Data System
