# Supplementary material for: Impact of prehospital medical evacuation (MEDEVAC) transport time on combat mortality in patients with non-compressible torso injury and traumatic amputations: a retrospective study
Source: Mil Med Res. 2018 Jun 30;5:22. doi: 10.1186/s40779-018-0169-2 (PMC6032797; doi:10.1186/s40779-018-0169-2)
Supplement: Supplementary file 1 — List of ICD-9 and Abbreviated Injury Scale (AIS) codes used for query. (DOCX 20 kb) [file 40779_2018_169_MOESM1_ESM.docx]

| **Additional File 1. List of ICD-9 and Abbreviated Injury Scale (AIS) codes used for query.** | | |
| --- | --- | --- |
| **I** | **Codes** | **Definition** |
| **Amputation** | 88.7.x | Traumatic Amputation of Arm and Hand |
|  | 896.x | Traumatic Amputation of Foot |
|  | 897.x | Traumatic Amputation of Leg(s) |
|  | | |
| **Non-compressible Torso Injury** | **Lung** |  |
|  | 32.xx | Resection; Partial Lobectomy; Lobectomy; Pneumonectomy; Incision in Chest Wall |
|  | 34.xx | Exploratory Throractomy; Insertion of Chest Tube; Thoracostomy (Needle); Thoracoscopy |
|  | 860.x | Traumatic Hemothorax; Traumatic Hemopneumothroax |
|  | 861.x | Lung Injury Unspecified; Lung Laceration |
|  | 862.x | Intrathoracic Injury Not Otherwise Specified; Multiple Intrathoracic Organ Injury |
|  | 441424.x | Lung Blast Injury (Overpressure/Exposure) |
|  | 442200.x | Hemothorax |
|  | **Torso Vascular Injury** |  |
|  | 39.xx | Angioplasty; Atherectomy; Repair of Blood Vessels with Tissue Graft or Patch Graft; Hemorrhage Control Not Otherwise Specified |
|  | 54.xx | Exploratory Laporatomy; Laporatomy or Reopening Operative Site; Laparotomy Not Elsewhere Classified |
|  | 901.x | Injury to Thoracic Aorta; Injury Innominate/Subclavian Artery & Vein; Injury to superior vena cava; Injury to Pulmonary Artery & Vein; Injury to Intercostal Artery & Vein; Injury to Internal Mammary Artery & Vein; Injury to Multiple Thoracic Blood Vessels; Injury to Azygos & Hemiaygos Veins; Injury Unspecified Thoracic Vessels |
|  | 902.x | Injury To Abdominal Aorta; Injury To Inferior Vena Cava; Injury to Hepatic Artery & Vein; Injury to Celiac/Mesentary Artery; Injury Superior Mesenteric Artery & Vein; Injury Splenic Vein; Injury Renal Artery & Vein; Injury Hypogastric Artery & Vein; Injury Iliac Artery & Vein; Injury Multiple Abdominal/Pelvic Vessels; Injury Specified Blood Vessels Of Abdominal/Vessels |
|  | 38.xx | Abdominal Vessel Resection/Anastomosis; Occlusion of Aorta; Ligation of Thoracic Vessel; Ligation of Abdominal Arteries or Veins; Occlusion of Aorta |
|  | 521104.x | Superior Mesenteric Artery Laceration/Perforation |
|  | 521108.x | Superior Mesenteric Artery Laceration with Major Rupture |
|  | 521202.x | Inferior Vena Cava Laceration/Perforation |
|  | 521206.x | Inferior Vena Cava with Major Rupture |
|  | 521299.x | Inferior Vena Cava NFS |
|  | 521606.x | Injury Abdominal/Pelvic Vessels; Major Rupture |
|  |  |  |
|  | 421004.x | Pulmonary Artery Laceration--Perforation or Major Rupture; Pulmonary Vein Laceration |
|  | 421008.x | Pulmonary Artery Major Laceration |
|  | 421099.x | Pulmonary Artery NFS |
|  | 421202.x | Pulmonary Vein Laceration |
|  | **Spleen** |  |
|  | 41.xx | Partial or Total Splenectomy |
|  | 544226.x | Spleen Laceration with Hilar Vessel Involvement & Major Devascularization |
|  | 544228.x | Spleen Laceration with Hilar Disruption& Total Devascularization; Avulsion |
|  | 865.xx | Spleen Injury Unspecified; Spleen Parenchyma Laceration-Grade 3; Spleen Massive Parencyma Disruption-Grade 4; Spleen Injury Unspecified |
|  | **Liver** |  |
|  | 50.xx | Partial Hepatectomy; Destruction Of Liver Lesion; Closure of Liver Laceration; Liver Repair |
|  | 864.xx | Liver Injury Unspecified; Liver Hematoma/Contusion; Liver Laceration-Minor, Moderate, Major |
|  | 541826.x | Liver Laceration with Parenchymal Disruption |
|  | 541828.x | Liver Laceration with Parenchymal Disruption; >3couinard's Segments with Single Lobe |
|  | 541830.x | Liver Laceration with Hepatic Avulsion |
|  | **Kidney** |  |
|  | 55.xx | Suture Kidney Laceration |
|  | 866.x | Kidney Laceration; Kidney Hematoma S/Rupture of Capsule; Open Kidney Laceration; Kidney Complicated-Disruption Of Parenchyma |
|  | 869.x | Injury to Unspecified Organs-Open |
|  | 541626.x | Kidney Laceration Extending Through Renal Cortex, Medulla, with Main Vessel Injury |
|  | 541628.x | Kidney Laceration with Hilum Avulsion; Total Destruction of Vascular System |
|  | **Pelvis** |  |
|  | 856100.x | Pelvic Ring Fracture - NFS |
|  | 856101.x | Pelvic Ring Fracture - Open NFS |
|  | 856151.x | Pelvic Ring Fracture - Posterior Arch, Isolated Fracture, Not Destroying Integrity Of Ring |
|  | 856152.x | Pelvic Ring Fracture -Open |
|  | 856162.x | Pelvic Ring Fracture - Incomplete Disruption Of Posterior Arch - Open |
|  | 856163.x | Pelvic Ring Fracture - Incomplete Disruption Of Posterior Arch, Blood Loss <20% By Volume |
|  | 856164.x | Pelvic Ring Fracture - Incomplete Disruption Of Posterior Arch, Blood Loss >20% By Volume |
|  | 856171.x | Pelvic Ring Fracture - Complete Disruption Of Posterior Arch & Pelvic Floor |
|  | 856173.x | Pelvic Ring Fracture - Complete Disruption Of Posterior Arch & Pelvic Floor Blood Loss >20% By Volume |
|  | 856174.x | Pelvic Ring Fracture - Complete Disruption Of Posterior Arch & Pelvic Floor |
